# The New York Institute of Stomatology—Offer of Prizes

**Published:** 1905-08

**Authors:** 


### THE NEW YORK INSTITUTE OF STOMATOLOGY—OFFER OF PRIZES.

Upon another page will be found the announcement of The New York Institute of Stomatology, offering two prizes for the best papers submitted on or before March 1, 1906. The first prize will be a gold medal and two hundred and fifty dollars. The second, a gold medal and one hundred dollars.

This generous offer, the first, it is believed, ever emanating from a local society, should receive an equally generous response. The time might profitably be extended, as the working months, so far as this country is concerned, will be practically limited to six, a time too short for serious research work.

This offer should stimulate the scientific mind of the country to compete upon a proper basis. The Fourth International Dental Congress made a somewhat similar offer, with the result that the response from this country was not at all representative, while all the papers from abroad were of a high order of merit. There is ample room for work in unsolved problems in dentistry, and we possess now a large body of educated young men who should make these the subject of careful research, and this is their golden opportunity. Will the response be equal to the occasion?

